# Development and validation of nomograms to predict survival of neuroendocrine carcinoma in genitourinary system: A population-based retrospective study

**DOI:** 10.1371/journal.pone.0303440

**Published:** 2024-06-05

**Authors:** Xiangnan Niu, Shiwei Sun, Wenjuan Fan, Peng Yue, Wei Yao, Yue Wang, Xiaoqian Deng, Fuyu Guo, Yangang Zhang

**Affiliations:** 1 Shanxi Bethune Hospital, Shanxi Academy of Medical Sciences, Tongji Shanxi Hospital, Third Hospital of Shanxi Medical University, Taiyuan, China; 2 Tongji Hospital, Tongji Medical College, Huazhong University of Science and Technology, Wuhan, China; 3 Department of Urology, Taiyuan Central Hospital of Shanxi Medical University, Taiyuan, China; 4 Department of Gynecology, Xian No. 1 Hospital, Xi’an, China; 5 Third Hospital of Shanxi Medical University, Shanxi Bethune Hospital, Shanxi Academy of Medical Sciences, Tongji Shanxi Hospital, Taiyuan, China; Affiliated Hospital of Nanjing University of Chinese Medicine: Jiangsu Province Academy of Traditional Chinese Medicine, CHINA

## Abstract

Neuroendocrine carcinoma (NEC) is a rare yet potentially perilous neoplasm. The objective of this study was to develop prognostic models for the survival of NEC patients in the genitourinary system and subsequently validate these models. A total of 7125 neuroendocrine neoplasm (NEN) patients were extracted. Comparison of survival in patients with different types of NEN before and after propensity score-matching (PSM). A total of 3057 patients with NEC, whose information was complete, were extracted. The NEC influencing factors were chosen through the utilization of the least absolute shrinkage and selection operator regression model (LASSO) and the Fine & Gary model (FGM). Furthermore, nomograms were built. To validate the accuracy of the prediction, the efficiency was verified using bootstrap self-sampling techniques and receiver operating characteristic curves. LASSO and FGM were utilized to construct three models. Confirmation of validation was achieved by conducting analyses of the area under the curve and decision curve. Moreover, the FGS (DSS analysis using FGM) model produced higher net benefits. To maximize the advantages for patients, the FGS model disregarded the influence of additional occurrences. Patients are expected to experience advantages in terms of treatment options and survival assessment through the utilization of these models.

## Introduction

Neuroendocrine neoplasm (NEN) is a rare but potentially dangerous type of neoplasm that originates in cells that produce hormones in the neuroendocrine system [1]. Neuroendocrine carcinoma (NEC), neuroendocrine tumor (NET), and mixed neuroendocrine-non-neuroendocrine neoplasm (MiNEN) are categorized according to their differentiation. NET are highly differentiated and have low malignancy, whereas NECs are poorly differentiated and have a high malignancy with a poor prognosis [2]. Neuroendocrine neoplasms (NENs) have the potential to develop in various regions of the anatomy, with the pancreas, lungs, and small intestine being the primary locations [3]. In comparison, NENs in genitourinary systems are relatively rare [4–6].

During embryonic development, both urinary and genital systems originate from the mesoderm. The urinary system consists of two kidneys, two ureters, the bladder, and the urethra, and its primary function is to filter blood and excrete waste. The genital system consists of the testes and epididymis (in males) or ovaries and fallopian tubes (in females), and its main function is reproduction. Due to their common origin and adjacent location, there is some connection between them, although they also differ in function [7].

Our research aims to explore the survival of patients with NEC of the genitourinary system and the factors that affect survival through algorithms such as the least absolute shrinkage and selection operator (LASSO), Cox proportional hazards model (CPH), and Fine & Gray model (FGM).

LASSO, initially suggested by Robert Tibshirani, is a powerful classifier employed in different classification or regression investigations for constructing machine learning models that yield accurate prediction outcomes. A recent study utilized LASSO to assess the complexity of retroperitoneal laparoscopic adrenalectomy and demonstrated a favorable predictive performance with an AUC of 0.787 [8].

The CPH model is a regression model with semiparametric characteristics. Survival analysis often utilizes this method as one of the most frequently employed techniques for analyzing multiple variables. The initial proposition of the FGM in 1999, aimed at analyzing the distribution of a rival risk, enables the elimination of interference caused by competing events in survival analysis, leading to a survival model with enhanced accuracy [9, 10].

In this study, data on NEC were gathered from the Surveillance, Epidemiology, and End Results (SEER) database, encompassing primary, clinical, and treatment information. The study examined the factors that impact both overall survival (OS) and disease-specific survival (DSS), ultimately developing predictive models. The aim of this study is to assist surgeons in tailoring treatment plans based on patients’ specific conditions, thereby improving patient outcomes and prolonging expected survival.

## Methods

### Data acquisition

**Fig 1:**
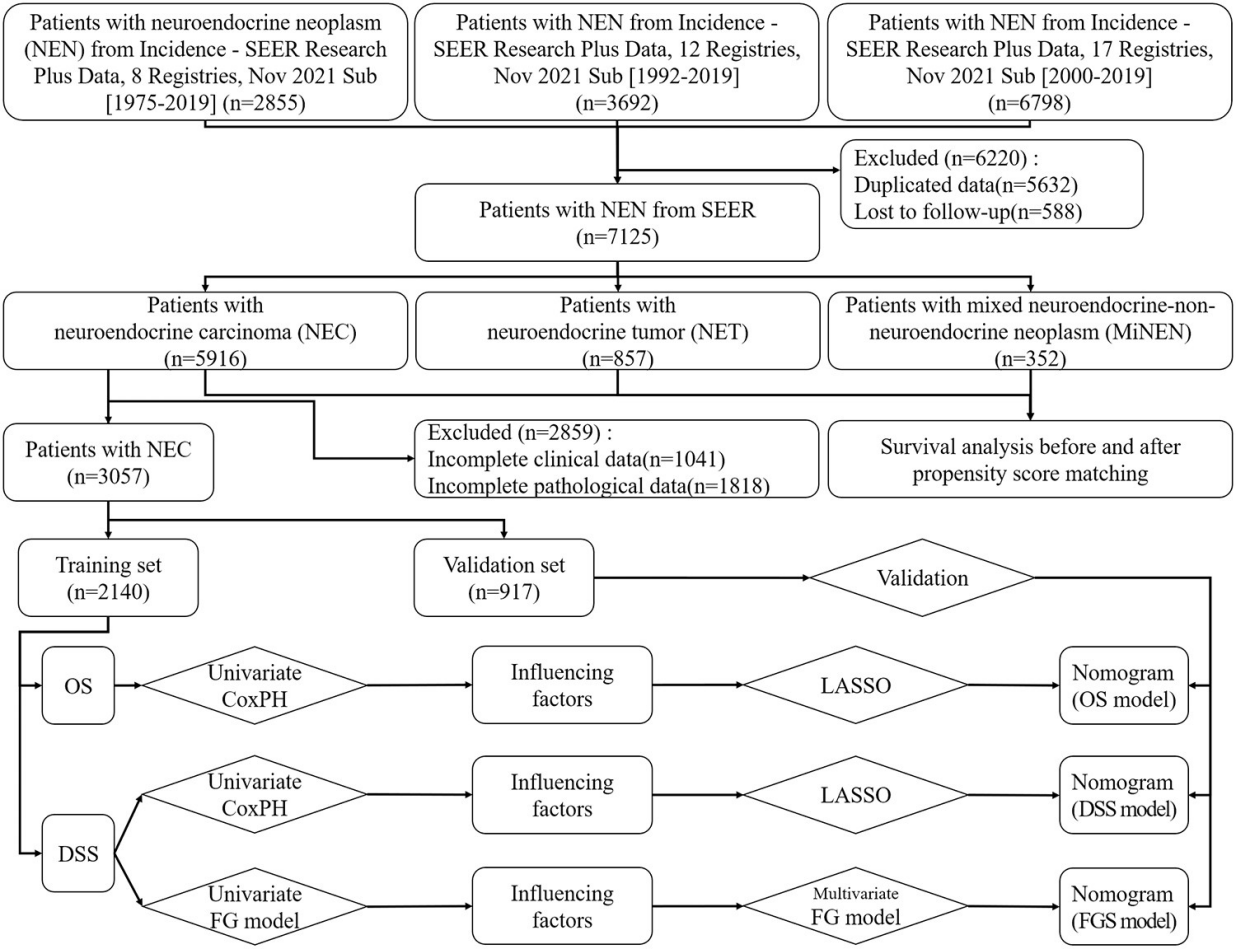
Study flowchart.

Information was obtained from the SEER database, specifically utilizing the SEER*Stat software version 8.4.0.1 that was made available on May 16, 2022. More than one-third of the American population is covered by this extensive database, which includes statistics on the occurrence and survival rates of cancer. Access to the SEER database is open to the public and does not necessitate approval from an ethics committee. From the three databases, we acquired a total of 13,345 patient data of NEN. (Specifically, the first database collected patient data from 8 Registries (each corresponding to one or multiple states) spanning from 1975 to 2019, comprising 2855 patients. The second database gathered data from 12 Registries spanning from 1992 to 2019, totaling 3692 patients. Meanwhile, the third database compiled data from 17 Registries spanning from 2000 to 2019, encompassing 6798 patients. As the SEER database evolved over time, newer databases incorporated data from more Registries, whereas older databases may include data from fewer Registries but potentially cover earlier periods. Thus, while there is some overlap between the datasets, they are not entirely identical.) The data underwent screening using the following criteria for inclusion and exclusion: Inclusion criteria: 1. NEN originating from the urinary system (Primary Site code = C64.9, C65.9, C66.9, C67.0, C67.1, C67.2, C67.3, C67.4, C67.5, C67.6, C67.7, C67.8, C67.9, C68.0, C68.8, C68.9), female genital system (Primary Site code = C51.0, C51.1, C51.2, C51.9, C52.9, C53.0, C53.1, C53.8, C53.9, C54.0, C54.1, C54.2, C54.3, C54.8, C54.9, C55.9, C56.9, C57.0, C57.2, C57.3, C57.4, C57.8, C57.9), and male genital system (Primary Site code = C60.1, C60.2, C60.9, C61.9, C62.0, C62.1, C62.9, C63.0, C63.2, C63.8, C63.9); 2. Clear pathological type (Histological code = 8002/3, 8013/3, 8041/3, 8042/3, 8043/3, 8044/3, 8045/3, 8240/3, 8241/3, 8242/3, 8243/3, 8244/3, 8245/3, 8246/3, 8247/3, 8249/3); 3. Data for follow-up is fully completed. The exclusion criteria are as follows: 1. Patient data duplication; 2. Insufficient information about the patient, including their age; 3.A lack of follow-up data. This study included a grand total of 7,125 individuals. Based on the histological codes, NENs were categorized as NEC, NET, or MiNEN (Fig 1).

### Clinicopathological characteristics

There are 261 various types of features included in the SEER database. We chose the age, gender, ethnicity, surgical procedure, lymph node dissection (LND), radiation therapy, chemotherapy, marital status, income, place of residence, stage, grade, and pathology as the primary observational characteristics. Deaths from any cause were considered events for OS, whereas tumor-related deaths were events for DSS.

### Statistical analysis

The information was processed utilizing R version 4.2.3 (Vienna Statistical Computing Foundation, Vienna, Austria). Since the continuous variables did not adhere to a normal distribution, we represented them using the median [interquartile range] and compared inter-group differences using the Mann-Whitney U test.. Generally, categorical variables are represented by frequencies and percentages (%), and we employed either the Pearson Chi-square test or Fisher’s exact test based on the magnitude of the cell’s minimum expected value. The survival of different NENs was compared using CPH and Kaplan-Meier (K-M) curves before and after propensity score matching (PSM). We utilized the “matchit” function from the “MatchIt” package, specifying the method as “nearest”. The variables included in the matching process were age, race, surgery, radiotherapy, chemotherapy, LND, marital status, income, residence, stage, and grade. By using Log-rank tests, different K-M curves were compared. In a ratio of 7:3, NEC-diagnosed patients were randomly divided into training and validation sets. To determine the factors that affected OS and DSS, univariate analyses using CPH and FGM were conducted. Subsequently, nomograms were created using the outcomes obtained from LASSO and FGM. Validation confirmed the efficacy of the models through the utilization of receiver operating characteristic (ROC) curves. Using bootstrap resampling and calibration curves, we assessed the models’ coherence. Using clinical decision curve analysis (DCA), the impact on patient benefits was evaluated. A significance level of P < 0.05 was set as the threshold for statistical significance.

## Results

### General information

This study included a grand total of 7125 individuals. S1 Table contains the essential data. The median age of the patients was 67 years. 3604 males (50.6%) and 3521 females (49.4%) were among them.NEC accounted for 83.0% of the total patients, while NET and MiNEN accounted for 12.0% and 5.0% respectively, with a total of 5916, 857, and 352 patients. The median OS was 15 months with a 95% confidence interval (CI) of 15–16. Additionally, the 1, 3, and 5 years OS were 56.0%, 33.6%, and 28.7% correspondingly. The DSS median was 19 months with a 95% CI of 18–20. Additionally, the DSS rates at 1, 3, and 5 years were 60.9%, 39.6%, and 35.5% correspondingly.

### Survival analysis between different types of NEN

**Fig 2:**
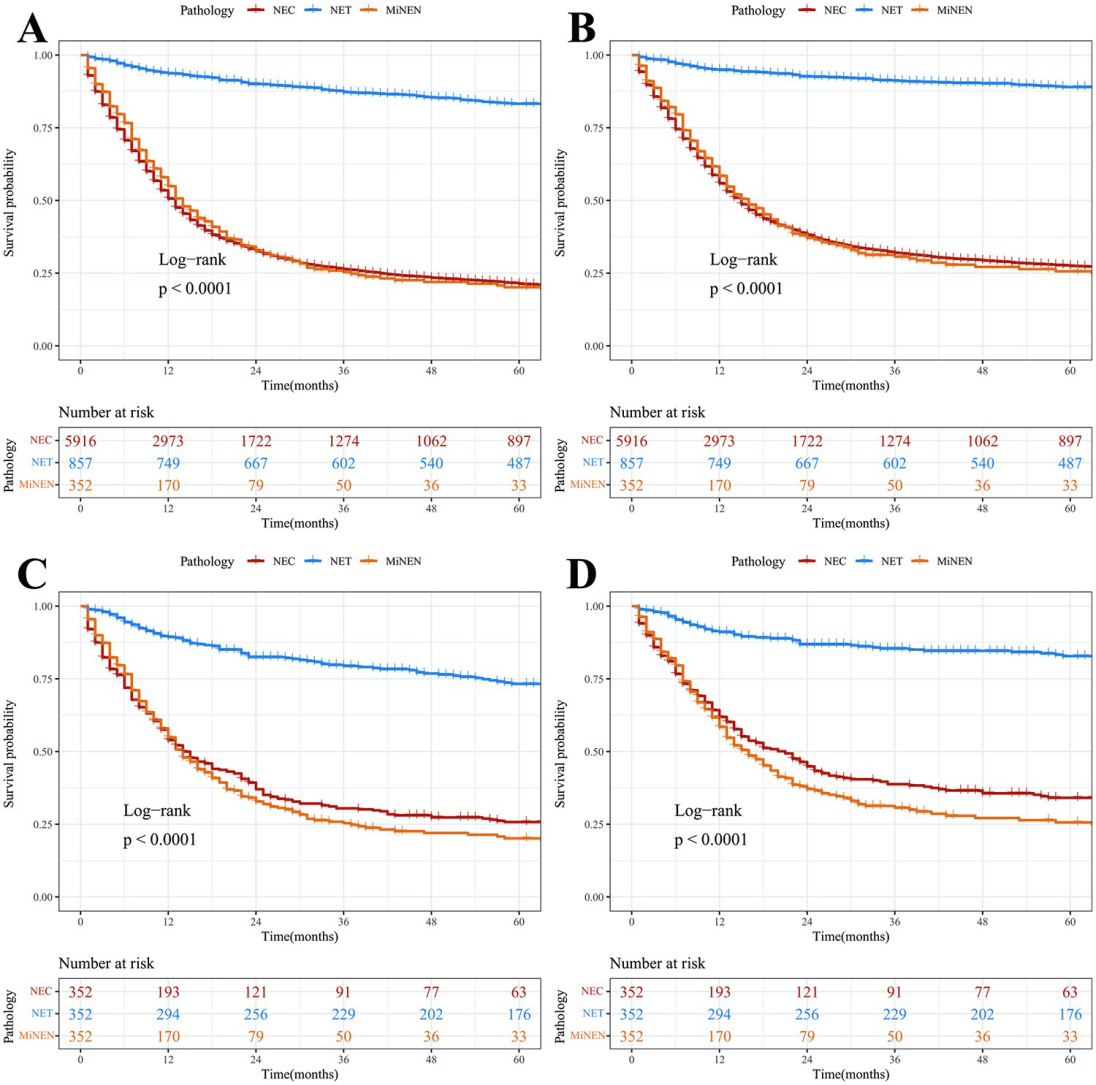
Survival rates prior and post- propensity score matching (PSM). (A) Overall survival [OS] before PSM; (B) Disease-specific survival (DSS) before PSM; (C) OS after PSM; (D). DSS after PSM.

The survival analysis of OS and DSS for all three pathological types of NEN is shown in Fig 2, indicating that NEC and MiNEN had significantly lower OS and DSS than NET. After propensity score matching (PSM), the results were similar to those before PSM. A total of 5916 patients had NEC. The K-M curves showed a difference in OS between different systems, but no significant difference was observed between different organs within each system (S1 Fig). After excluding incomplete patient data, 3057 patients left in further analysis (S2 and S3 Tables). The training and validation sets were randomly divided 7:3 with no statistical differences between the sets (Table 1).

**Table 1:** Baseline characteristics of the patients with NEC.

Variables	Total cohort	Sets		F/χ^2^	P
	(n = 3057)	Training (n = 2140)	Validation (n = 917)		
Age	69.0[57.0,78.0]	69.0[57.0,78.0]	69.0[58.0,77.0]	0.164	0.870
Sex				0.492	0.483
Male	1654(54.1)	1149(69.5)	505(30.5)		
Female	1403(45.9)	991(70.6)	412(29.4)		
Race				1.672	0.196
White	2529(82.7)	1758(69.5)	771(30.5)		
Others	528(17.3)	382(72.3)	146(27.7)		
System/Organ				11.136	0.267
Urinary System	1650(54.0)	1166(70.7)	484(29.3)		
Bladder	1525(49.9)	1077(70.6)	448(29.4)		
Kidney	85(2.8)	58(68.2)	27(31.8)		
Ureter	40(1.3)	31(77.5)	9(22.5)		
Female Genital System	1026(33.6)	714(69.6)	312(30.4)		
Uterus	738(24.1)	518(70.2)	220(29.8)		
Ovary	219(7.2)	146(66.7)	73(33.3)		
Vagina	58(1.9)	45(77.6)	13(22.4)		
Vulva	11(0.4)	5(45.5)	6(54.5)		
Male Genital System	381(12.5)	260(68.2)	121(31.8)		
Prostate	378(12.4)	258(68.3)	120(31.7)		
Testis	2(0.1)	2(100.0)	0(0.0)		
Penis	1(0.0)	0(0.0)	1(100.0)		
Pathology				0.349	0.84
SCNEC	1856(60.7)	1301(70.1)	555(29.9)		
LCNEC	180(5.9)	129(71.7)	51(28.3)		
NOS	1021(33.4)	710(69.5)	311(30.5)		
Surgery				0.972	0.324
None	756(24.7)	540(71.4)	216(28.6)		
Yes	2301(75.3)	1600(69.5)	701(30.5)		
Lymph node dissection				1.720	0.19
None	2236(73.1)	1580(70.7)	656(29.3)		
Yes	821(26.9)	560(68.2)	261(31.8)		
Radiotherapy				0.464	0.496
None/Unknown	2070(67.7)	1441(69.6)	629(30.4)		
Yes	987(32.3)	699(70.8)	288(29.2)		
Chemotherapy				0.077	0.782
None/Unknown	1049(34.3)	731(69.7)	318(30.3)		
Yes	2008(65.7)	1409(70.2)	599(29.8)		
Marital status				0.319	0.572
Married	1780(58.2)	1239(69.6)	541(30.4)		
Single	1277(41.8)	901(70.6)	376(29.4)		
Income				0.109	0.742
High	1714(56.1)	1204(70.2)	510(29.8)		
Low	1343(43.9)	936(69.7)	407(30.3)		
Residence				0.113	0.737
Urban	2706(88.5)	1897(70.1)	809(29.9)		
Rural	351(11.5)	243(69.2)	108(30.8)		
Stage				0.257	0.879
Localized	1152(37.7)	802(69.6)	350(30.4)		
Regional	835(27.3)	590(70.7)	245(29.3)		
Distant	1070(35.0)	748(69.9)	322(30.1)		
Grade				1.967	0.579
Grade I	37(1.2)	25(67.6)	12(32.4)		
Grade II	63(2.1)	49(77.8)	14(22.2)		
Grade III	1693(55.4)	1181(69.8)	512(30.2)		
Grade IV	1264(41.3)	885(70.0)	379(30.0)		

### Univariate analysis

Univariate CPH analysis showed the impact of various variables on OS and DSS (S2 Fig). The factors significantly influencing OS include age, sex, system, surgery, LND, radiotherapy, chemotherapy, marital status, stage, and grade (S3 Fig). While the factors affecting DSS include age, sex, system, surgery, LND, radiotherapy, chemotherapy, marital status, stage, and grade (S4 Fig).

Univariate FGM analysis showed that the main factors affecting DSS were age (F = 141.056, P<0.001), system (F = 30.059, P<0.001), surgery (F = 66.819, P<0.001), LND (F = 63.096, P<0.001), stage (F = 238.349, P<0.001), grade (F = 20.704, P<0.001), radiotherapy (F = 5.182, P = 0.023), and marital status (F = 4.87, P = 0.027) (S5 Fig).

#### LASSO

Based on the results of univariate analysis, variables with P>0.2, which are deemed unrelated, were excluded. The remaining variables were included in the subsequent analysis. Using dummy variables, we converted multi-categorical variables into binary-categorical variables, and the final variable assignments are shown in S4 Table. Through a 10-fold cross-validation process, we performed LASSO with a log(lambda) value of the harmonic parameter for all variables (S6 Fig). After LASSO for both OS and DSS, eight influencing factors were selected for each model: age, surgery, LND, chemotherapy, stage = localized, stage = distant, grade = grade I, and grade = grade II (S5 Table).

#### Nomogram

**Fig 3:**
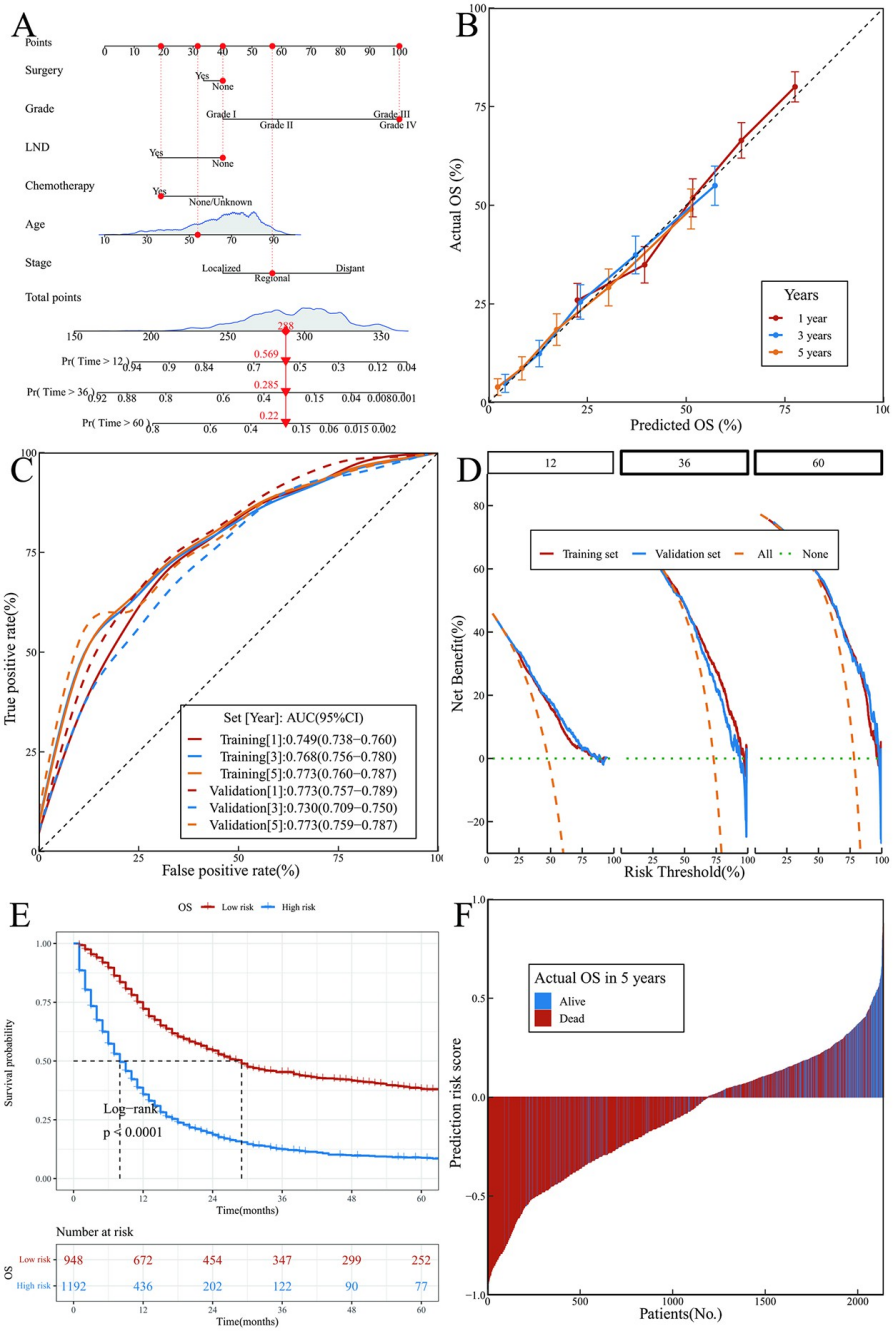
OS model and its performance. (A) Nomogram; (B) Calibration curves; (C) ROC curves; (D)DCA curves; (E) K-M curves of predicted risk subgroups; (F) Predicted risk scores for each patient.

**Fig 4:**
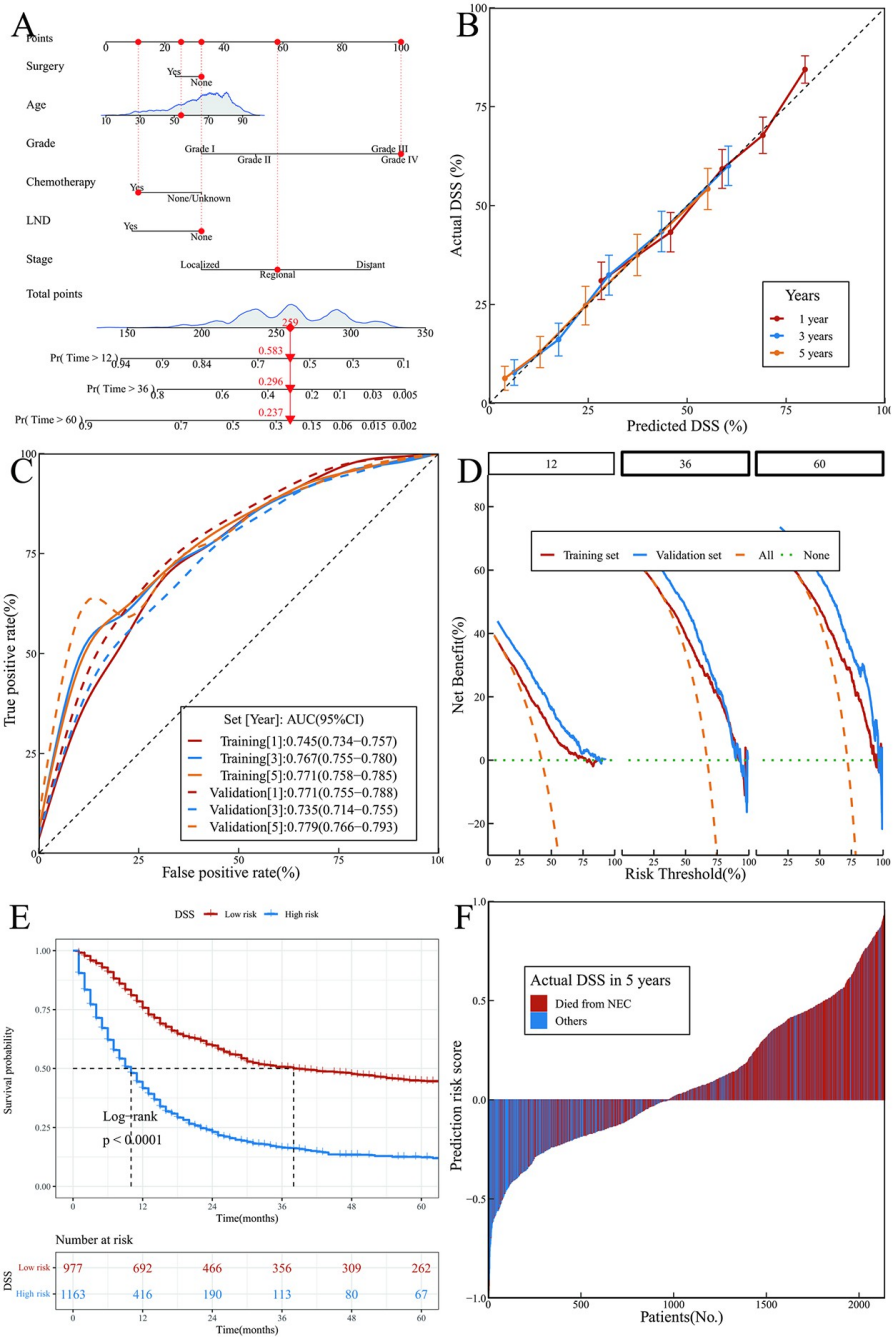
DSS model and its performance. (A) Nomogram; (B) Calibration curves; (C) ROC curves; (D)DCA curves; (E) K-M curves of predicted risk subgroups; (F) Predicted risk scores for each patient.

**Fig 5:**
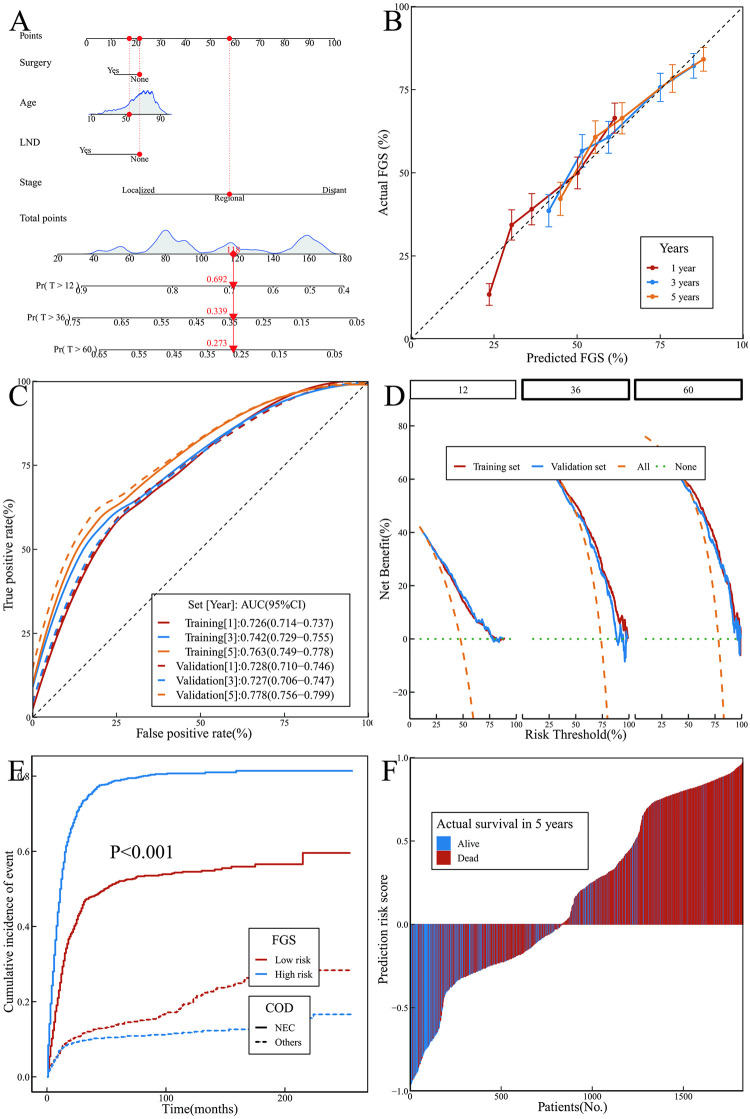
FGS model and its performance. (A) Nomogram; (B) Calibration curves; (C) ROC curves; (D)DCA curves; (E) K-M curves of predicted risk subgroups; (F) Predicted risk scores for each patient.

Nomograms depict the OS and DSS models (Figs 3A and 4A) constructed using LASSO. Furthermore, a multivariate FGM was built through stepwise regression to acquire the DSS predicted by FGM (FGS) and visualized using a nomogram (Fig 5A). The nomograms were utilized by adding up the points for each index of the patient to determine the total points. The total points line contained the corresponding risk value for the patient’s likelihood of experiencing an event. In the case of a patient identified as 815441, a Caucasian woman aged 54 who is married and has NEC in the uterus, at a regional stage with grade 4, without any surgical treatment or LND, but received chemotherapy and radiotherapy, the OS analysis indicated scores of 40, 100, 40, 19, 32, and 57 for each indicator, resulting in a total score of 288 points. The OS for 1, 3, and 5 years were 56.9%, 28.5%, and 22.0%, respectively. DSS achieved a total score of 259, with individual scores of 32, 26, 100, 11, 32, and 58 for each indicator. At 1, 3, and 5 years, the DSS rates were 58.3%, 29.6%, and 23.7% in that order. The FGS had scores of 21, 18, 21, and 58 for each indicator, resulting in a total score of 118 points.The FGS rates at 1, 3, and 5 years were 69.2%, 33.9%, and 27.3% correspondingly.

### Validation and performance of the nomograms

The OS model was internally validated through 1000 resampling iterations. The calibration curve aligned nicely with the perfect curve (Fig 3B), demonstrating strong agreement between the model’s projected likelihood and real circumstances. Based on the results of fitting the model, a ROC curve was generated. For the training and validation sets, the AUCs shown as follows: 0.749 (95%CI: 0.738–0.760) and 0.773 (95%CI: 0.757–0.789) for 1-year OS, 0.768 (95%CI: 0.756–0.780) and 0.730 (95%CI: 0.709–0.750) for 3-year OS, and 0.773 (95%CI: 0.760–0.787) and 0.773 (95%CI: 0.759–0.787) for 5-year OS, respectively. These results indicate a strong predictive capability of the model (Fig 3C). According to DCA, the model demonstrated the greatest overall advantage of around 17%, 50%, and 59% for individuals after 1, 3, and 5 years, correspondingly (Fig 3D). The threshold corresponding to the point on the ROC curve that maximizes the sum of sensitivity and specificity will serve as the cut-off value. Based on this value (214.90), patients will be categorized into low-risk and high-risk groups. The low-risk group had a median OS of 29 months (95%CI 25–32), while that of the high-risk group was 8 months (95%CI 8–9) (Fig 3E). With a specificity of 0.718, precision of 0.864, recall of 0.658, and F1-score of 0.747, the model was highly accurate (Fig 3F).

The DSS model was validated using the same verification methods as that of the OS model, confirming its good predictive performance. At 1, 3, and 5 years, the net benefits to patients can be increased by 15%, 46%, and 53%, respectively. Using 218.00 as the cut-off value, significant differences in survival were observed between the high-risk and low-risk groups. A combination of its specificity (0.627), precision (0.737), recall (0.649), and F1-score (0.681) showed its high prediction accuracy. (Fig 4B–4F).

The internal validation of the FGS model was conducted by performing 1000 resampling iterations. The calibration curves exhibited a good fit with the ideal curves (Fig 5B). The AUCs for 1-year FGS in the training and validation sets were 0.726 (95%CI 0.714–0.737) and 0.728 (95%CI 0.710–0.746), respectively. For 3-year FGS, the AUCs were 0.742 (95%CI 0.729–0.755) and 0.727 (95%CI 0.706–0.747). Additionally, for 5-year FGS, the AUCs were 0.763 (95%CI 0.749–0.778) and 0.778 (95%CI 0.756–0.799) (Fig 5C). According to DCA, the model showed a rise in patient net benefits of around 16%, 52%, and 59% after 1, 3, and 5 years, respectively (as shown in Fig 5D). The threshold corresponding to the point on the ROC curve that maximizes the sum of sensitivity and specificity will serve as the cut-off value. Based on this value (101.03), patients will be categorized into low-risk and high-risk groups. The model’s risk grouping predicted a notable contrast in survival rates for patients who succumbed to NEC between the low- and high-risk categories (F = 200.8, P<0.001) (Fig 5E). With a specificity of 0.677, precision of 0.835, recall of 0.629, and F1-score of 0.718, the model was highly accurate (Fig 5F).

Based on the DCA findings, it is evident that the FGS model outperforms the DSS models relying on LASSO in terms of predictive accuracy and can yield greater advantages for patients.

## Discussion

NEN is a rare type of cancer originating from neuroendocrine cells and can occur in any part of the body, including the lungs, stomach, pancreas, small intestine, colon, and rectum [1]. The clinical symptoms of NEN vary depending on the site of occurrence and type of hormone secretion, including abdominal pain, diarrhea, vomiting, weight loss, and flushing [11, 12].

Recently, the classification and diagnostic criteria for NEN have been updated continuously. In 2017, the World Health Organization (WHO) released classification criteria that merged the original NET and NEC into NEN and further divided it into three subtypes, G1, G2, and G3, based on histological and molecular genetic characteristics [13]. Then WHO proposed a universal differentiation and proliferation grading system for neuroendocrine tumors in 2022 [1].

In the genitourinary system, clear cell carcinoma (in the kidney), transitional cell carcinoma (in the bladder/ureter), and squamous cell carcinoma (in the uterus) are more common pathological types [6]. NEN can occur in any part of the genitourinary system, including the kidneys, bladder, prostate, testicles, and ovaries, with the bladder, prostate, and uterus being the most common sites. However, NEN has a significantly lower incidence rate than other types of genitourinary system tumors. NEC has a similarly low incidence rate, but a faster growth rate and a higher tendency to metastasize and recur [5, 14].

Based on the SEER data of patients with NEN in the genitourinary system, this study compared the survival of different types of NEN through PSM. CPH analysis was used to identify the factors affecting the survival of patients with NEC. LASSO combined with ten-fold cross-validation was used to analyze patient prognosis and construct OS and DSS prediction models that included age, surgery, LND, chemotherapy, stage, and grade. Univariate FGM and stepwise regression analyses were used to construct a model that included age, surgery, LND, and stage. This study found that FGM could provide more significant benefits to patients. Furthermore, we found that there were differences in the survival of patients with NEC between different systems, with female genital system NEC having better survival outcomes than urinary and male genital system NECs. However, there was a similarity in survival outcomes among patients with NEC in different organs within each system.

Age has a prognostic impact on almost all tumors. Elderly individuals often experience inadequate nutritional conditions and reduced tolerance. However, they often have higher staging at diagnosis. Stage and grade significantly affect patient prognosis [15–17]. A higher stage or grade usually indicates poorer differentiation, greater malignancy, and tremendous potential for metastasis, which can affect patient survival [18, 19]. As a malignant wasting disease, a higher stage or grade of NEC can cause greater consumption, burden, and impact on the body [20].

Treatment also affects the prognosis [21]. The prognosis of the treated patients in our study was superior to that of the untreated patients. Both surgery and chemotherapy play a crucial role in enhancing patient survival, with early surgical intervention being particularly beneficial. Radiotherapy also plays an important role [22]. Research on targeted therapy for NEC of the genitourinary system is relatively scarce. Some studies have proposed ubiquitin carboxy-terminal hydrolase L1 (UCHL1), a deubiquitinating enzyme, as a therapeutic target for it [23]. Additionally, in NEC of other systems such as the pancreas and thyroid, RET has been regarded as an effective predictive marker, and RET-targeting tyrosine kinase inhibitors are considered to have potential therapeutic effects [24, 25]. The metastatic pathways of NEC can be through the lymphatic system, blood circulation, and direct infiltration [1, 26]. The lymphatic system is the most common route of metastasis. Cancer cells can invade the surrounding lymph nodes and spread to other areas of the lymphatic system. As NEC is highly malignant and tends to metastasize early, LND can effectively slow down or even block the lymphatic metastasis pathway of NEC, thereby slowing disease progression and improving patient survival. Some scholars have proposed that complete surgical resection is considered to improve prognosis regardless of the NEN histological type, while LND is necessary for complete resection. This view has gradually reached consensus through extensive clinical practice and research [19, 27].

Currently, there are few studies on the survival of NEC patients in the genitourinary system. The uniqueness of this research lies in analyzing the factors affecting the survival of NEC in the genitourinary system through LASSO and FGM analyses while excluding interference from other events. The OS and DSS prediction models were established and validated using DCA analysis. According to the FGS model, which excludes interference from other factors, DCA could bring a greater net benefit to patients.

The study had several limitations: 1) Patients were exposed to different living environments and treatment conditions over a long period of time; 2) The study only employed LASSO and FGM techniques. In the next step of the research, other machine learning algorithms, such as random forest, were compared to obtain the best model.

## Conclusion

To summarize, the age, surgical procedure, LND, chemotherapy, stage, and grade had a significant impact on OS and DSS of individuals diagnosed with NEC in the genitourinary tract. The predictive performance of the models, which were developed using LASSO and FGM, was excellent. FGM can provide significant net benefits to patients. Through this model, it is possible to effectively evaluate the survival expectancy of patients with NEC in the genitourinary system, facilitate personalized treatment design, improve survival expectancy, and benefit patients.

## Supporting information

S1 Table Baseline characteristics of the patients with NEN. (DOCX)

S2 Table Comparison of baseline characteristics of OS for patients with NEC. (DOCX)

S3 Table Comparison of baseline characteristics of DSS for patients with NEC. (DOCX)

S4 Table Variable assignments. (DOCX)

S5 Table Risk factors selected by LASSO. (DOCX)

S1 Fig Survival of patient with NEC in different systems, organs, and pathology. (TIF)

S2 Fig Forest plot of univariate Cox proportional hazard models. (TIF)

S3 Fig Kaplan—Meier curves of OS for patients. (TIF)

S4 Fig Kaplan—Meier curves of DSS for patients. (TIF)

S5 Fig Fine-Grey models for patients. (TIF)

S6 Fig The process of LASSO. (TIF)
