# Supplementary material for: Development and validation of nomograms to predict survival of neuroendocrine carcinoma in genitourinary system: A population-based retrospective study
Source: PLoS One. 2024 Jun 5;19(6):e0303440. doi: 10.1371/journal.pone.0303440 (PMC11152281; doi:10.1371/journal.pone.0303440)
Supplement: S1 Table — (DOCX) [file pone.0303440.s001.docx]

# S1 Table. Baseline characteristics of the patients with NEN.

| Variables | Total cohort | Overall survival | | F/χ^2^ | P |
| --- | --- | --- | --- | --- | --- |
|  | (n=7125) | Dead(n=5146) | Alive(n=1979) |  |  |
| Age(year) | 67.0[54.0,77.0] | 70.0[58.0,79.0] | 58.0[44.0,69.0] | 23.864 | <0.001 |
| Sex |  |  |  | 240.327 | <0.001 |
| Male | 3604(50.6) | 2896(80.4) | 708(19.6) |  |  |
| Female | 3521(49.4) | 2250(63.9) | 1271(36.1) |  |  |
| Race |  |  |  | 19.261 | <0.001 |
| White | 5871(82.4) | 4286(73.0) | 1585(27.0) |  |  |
| Black | 664(9.3) | 485(73.0) | 179(27.0) |  |  |
| Asian or Pacific Islander | 520(7.3) | 336(64.6) | 184(35.4) |  |  |
| American Indian/Alaska Native | 41(0.6) | 34(82.9) | 7(17.1) |  |  |
| Unknown | 29(0.4) | 5(17.2) | 24(82.8) |  |  |
| System/Organ |  |  |  | 654.909 | <0.001 |
| Urinary System | 3178(44.6) | 2459(77.4) | 719(22.6) |  |  |
| Bladder | 2762(38.8) | 2179(78.9) | 583(21.1) |  |  |
| Kidney | 295(4.1) | 195(66.1) | 100(33.9) |  |  |
| Ureter | 81(1.1) | 58(71.6) | 23(28.4) |  |  |
| Other Urinary Organs | 40(0.6) | 27(67.5) | 13(32.5) |  |  |
| Female Genital System | 2758(38.7) | 1701(61.7) | 1057(38.3) |  |  |
| Uterus | 1471(20.6) | 1041(70.8) | 430(29.2) |  |  |
| Ovary | 1089(15.3) | 499(45.8) | 590(54.2) |  |  |
| Vagina | 119(1.7) | 104(87.4) | 15(12.6) |  |  |
| Vulva | 39(0.5) | 30(76.9) | 9(23.1) |  |  |
| Other Female Genital Organs | 40(0.6) | 27(67.5) | 13(32.5) |  |  |
| Male Genital System | 1189(16.7) | 986(82.9) | 203(17.1) |  |  |
| Prostate | 1113(15.6) | 963(86.5) | 150(13.5) |  |  |
| Testis | 60(0.8) | 13(21.7) | 47(78.3) |  |  |
| Penis | 3(0.0) | 3(100.0) | 0(0.0) |  |  |
| Other Male Genital Organs | 13(0.2) | 7(53.8) | 6(46.2) |  |  |
| Pathology |  |  |  | 1049.21 | <0.001 |
| NEC | 5916(83.0) | 4680(79.1) | 1236(20.9) |  |  |
| SCNEC | 3995(56.1) | 3149(78.8) | 846(21.2) |  |  |
| LCNEC | 285(4.0) | 199(69.8) | 86(30.2) |  |  |
| NOS | 1636(23.0) | 1332(81.4) | 304(18.6) |  |  |
| NET | 857(12.0) | 224(26.1) | 633(73.9) |  |  |
| MiNEN | 352(4.9) | 242(68.8) | 110(31.2) |  |  |
| Diameter(mm) | 50.0[30.0,79.0] | 51.0[35.0,82.0] | 40.0[20.0,69.0] | 10.641 | <0.001 |
| T stage |  |  |  | 835.21 | <0.001 |
| 1 | 2173(30.5) | 1096(50.4) | 1077(49.6) |  |  |
| 2 | 1776(24.9) | 1307(73.6) | 469(26.4) |  |  |
| 3 | 1929(27.1) | 1646(85.3) | 283(14.7) |  |  |
| 4 | 1153(16.2) | 1013(87.9) | 140(12.1) |  |  |
| X | 94(1.3) | 84(89.4) | 10(10.6) |  |  |
| N stage |  |  |  | 138.28 | <0.001 |
| 0 | 5165(72.5) | 3538(68.5) | 1627(31.5) |  |  |
| 1 | 1535(21.5) | 1236(80.5) | 299(19.5) |  |  |
| 2 | 275(3.9) | 240(87.3) | 35(12.7) |  |  |
| 3 | 56(0.8) | 48(85.7) | 8(14.3) |  |  |
| X | 94(1.3) | 84(89.4) | 10(10.6) |  |  |
| M stage |  |  |  | 536.983 | <0.001 |
| 0 | 4472(62.8) | 2808(62.8) | 1664(37.2) |  |  |
| 1 | 2385(33.5) | 2116(88.7) | 269(11.3) |  |  |
| X | 268(3.8) | 222(82.8) | 46(17.2) |  |  |
| Surgery |  |  |  | 268.85 | <0.001 |
| None | 1923(27.0) | 1634(85.0) | 289(15.0) |  |  |
| Yes | 4485(62.9) | 2899(64.6) | 1586(35.4) |  |  |
| Unknown | 717(10.1) | 613(85.5) | 104(14.5) |  |  |
| LND |  |  |  | 205.145 | <0.001 |
| None | 5238(73.5) | 3960(75.6) | 1278(24.4) |  |  |
| Yes | 1321(18.5) | 736(55.7) | 585(44.3) |  |  |
| Unknown | 566(7.9) | 450(79.5) | 116(20.5) |  |  |
| Radiotherapy |  |  |  | 22.887 | <0.001 |
| None/Unknown | 5092(71.5) | 3596(70.6) | 1496(29.4) |  |  |
| Yes | 2033(28.5) | 1550(76.2) | 483(23.8) |  |  |
| Chemotherapy |  |  |  | 21.084 | <0.001 |
| None/Unknown | 3121(43.8) | 2168(69.5) | 953(30.5) |  |  |
| Yes | 4004(56.2) | 2978(74.4) | 1026(25.6) |  |  |
| Marital status |  |  |  | 3.381 | 0.066 |
| Married | 3953(55.5) | 2836(71.7) | 1117(28.3) |  |  |
| Single | 2843(39.9) | 2097(73.8) | 746(26.2) |  |  |
| Unknown | 329(4.6) | 213(64.7) | 116(35.3) |  |  |
| Income |  |  |  | 11.453 | 0.001 |
| High | 3984(55.9) | 2793(70.1) | 1191(29.9) |  |  |
| Low | 2922(41.0) | 2157(73.8) | 765(26.2) |  |  |
| Unknown | 219(3.1) | 196(89.5) | 23(10.5) |  |  |
| Residence |  |  |  | 7.149 | 0.007 |
| Urban | 6096(85.6) | 4335(71.1) | 1761(28.9) |  |  |
| Rural | 793(11.1) | 600(75.7) | 193(24.3) |  |  |
| Unknown | 236(3.3) | 211(89.4) | 25(10.6) |  |  |
| Stage |  |  |  | 701.196 | <0.001 |
| Localized | 2869(40.3) | 1618(56.4) | 1251(43.6) |  |  |
| Regional | 1777(24.9) | 1328(74.7) | 449(25.3) |  |  |
| Distant | 2385(33.5) | 2116(88.7) | 269(11.3) |  |  |
| Unstaged | 94(1.3) | 84(89.4) | 10(10.6) |  |  |
| Grade |  |  |  | 475.966 | <0.001 |
| Grade I | 222(3.1) | 44(19.8) | 178(80.2) |  |  |
| Grade II | 157(2.2) | 79(50.3) | 78(49.7) |  |  |
| Grade III | 2217(31.1) | 1660(74.9) | 557(25.1) |  |  |
| Grade IV | 1762(24.7) | 1472(83.5) | 290(16.5) |  |  |
| Unknown | 2767(38.8) | 1891(68.3) | 876(31.7) |  |  |
| Metastasis(bone) |  |  |  | 93.576 | <0.001 |
| None | 4129(58.0) | 2694(65.2) | 1435(34.8) |  |  |
| Yes | 562(7.9) | 481(85.6) | 81(14.4) |  |  |
| Unknown | 2434(34.2) | 1971(81.0) | 463(19.0) |  |  |
| Metastasis(brain) |  |  |  | 15.348 | <0.001 |
| None | 4617(64.8) | 3108(67.3) | 1509(32.7) |  |  |
| Yes | 68(1.0) | 61(89.7) | 7(10.3) |  |  |
| Unknown | 2440(34.2) | 1977(81.0) | 463(19.0) |  |  |
| Metastasis(liver) |  |  |  | 111.986 | <0.001 |
| None | 4177(58.6) | 2718(65.1) | 1459(34.9) |  |  |
| Yes | 516(7.2) | 455(88.2) | 61(11.8) |  |  |
| Unknown | 2432(34.1) | 1973(81.1) | 459(18.9) |  |  |
| Metastasis(lung) |  |  |  | 67.824 | <0.001 |
| None | 4336(60.9) | 2860(66.0) | 1476(34.0) |  |  |
| Yes | 336(4.7) | 295(87.8) | 41(12.2) |  |  |
| Unknown | 2453(34.4) | 1991(81.2) | 462(18.8) |  |  |
| Metastasis(distant lymph nodes) |  |  |  | 16.421 | <0.001 |
| None | 3814(53.5) | 2390(62.7) | 1424(37.3) |  |  |
| Yes | 232(3.3) | 176(75.9) | 56(24.1) |  |  |
| Unknown | 3079(43.2) | 2580(83.8) | 499(16.2) |  |  |
| Metastasis(others sites) |  |  |  | 404.034 | <0.001 |
| None | 3714(52.1) | 2308(62.1) | 1406(37.9) |  |  |
| Yes | 1421(19.9) | 1291(90.9) | 130(9.1) |  |  |
| Unknown | 1990(27.9) | 1547(77.7) | 443(22.3) |  |  |
