# Supplementary material for: Development and validation of nomograms to predict survival of neuroendocrine carcinoma in genitourinary system: A population-based retrospective study
Source: PLoS One. 2024 Jun 5;19(6):e0303440. doi: 10.1371/journal.pone.0303440 (PMC11152281; doi:10.1371/journal.pone.0303440)
Supplement: S2 Table — (DOCX) [file pone.0303440.s002.docx]

# S2 Table. Comparison of baseline characteristics of overall survival (OS) for patients with Neuroendocrine Carcinoma (NEC).

| Variables | Total cohort | Overall survival | | F/χ^2^ | P |
| --- | --- | --- | --- | --- | --- |
|  | (n=3057) | Dead(n=2376) | Alive(n=681) |  |  |
| Age | 69.0[57.0,78.0] | 70.0[59.0,79.0] | 63.0[50.0,72.0] | 11.862 | <0.001 |
| Sex |  |  |  | 45.648 | <0.001 |
| Male | 1654(54.1) | 1363(82.4) | 291(17.6) |  |  |
| Female | 1403(45.9) | 1013(72.2) | 390(27.8) |  |  |
| Race |  |  |  | 0.151 | 0.698 |
| White | 2529(82.7) | 1969(77.9) | 560(22.1) |  |  |
| Others | 528(17.3) | 407(77.1) | 121(22.9) |  |  |
| System/Organ |  |  |  | 62.234 | <0.001 |
| Urinary System | 1650(54.0) | 1305(79.1) | 345(20.9) |  |  |
| Bladder | 1525(49.9) | 1217(79.8) | 308(20.2) |  |  |
| Kidney | 85(2.8) | 62(72.9) | 23(27.1) |  |  |
| Ureter | 40(1.3) | 26(65.0) | 14(35.0) |  |  |
| Female Genital System | 1026(33.6) | 728(71.0) | 298(29.0) |  |  |
| Uterus | 738(24.1) | 515(69.8) | 223(30.2) |  |  |
| Ovary | 219(7.2) | 154(70.3) | 65(29.7) |  |  |
| Vagina | 58(1.9) | 50(86.2) | 8(13.8) |  |  |
| Vulva | 11(0.4) | 9(81.8) | 2(18.2) |  |  |
| Male Genital System | 381(12.5) | 343(90.0) | 38(10.0) |  |  |
| Prostate | 378(12.4) | 342(90.5) | 36(9.5) |  |  |
| Testis | 2(0.1) | 0(0.0) | 2(100.0) |  |  |
| Penis | 1(0.0) | 1(100.0) | 0(0.0) |  |  |
| Pathology |  |  |  | 6.236 | 0.044 |
| SCNEC | 1856(60.7) | 1426(76.8) | 430(23.2) |  |  |
| LCNEC | 180(5.9) | 132(73.3) | 48(26.7) |  |  |
| NOS | 1021(33.4) | 818(80.1) | 203(19.9) |  |  |
| Surgery |  |  |  | 59.262 | <0.001 |
| None | 756(24.7) | 664(87.8) | 92(12.2) |  |  |
| Yes | 2301(75.3) | 1712(74.4) | 589(25.6) |  |  |
| Lymph node dissection |  |  |  | 191.505 | <0.001 |
| None | 2236(73.1) | 1879(84.0) | 357(16.0) |  |  |
| Yes | 821(26.9) | 497(60.5) | 324(39.5) |  |  |
| Radiotherapy |  |  |  | 6.838 | 0.009 |
| None/Unknown | 2070(67.7) | 1637(79.1) | 433(20.9) |  |  |
| Yes | 987(32.3) | 739(74.9) | 248(25.1) |  |  |
| Chemotherapy |  |  |  | 58.699 | <0.001 |
| None/Unknown | 1049(34.3) | 899(85.7) | 150(14.3) |  |  |
| Yes | 2008(65.7) | 1477(73.6) | 531(26.4) |  |  |
| Marital status |  |  |  | 1.860 | 0.173 |
| Married | 1780(58.2) | 1368(76.9) | 412(23.1) |  |  |
| Single | 1277(41.8) | 1008(78.9) | 269(21.1) |  |  |
| Income |  |  |  | 2.007 | 0.157 |
| High | 1714(56.1) | 1316(76.8) | 398(23.2) |  |  |
| Low | 1343(43.9) | 1060(78.9) | 283(21.1) |  |  |
| Residence |  |  |  | 0.961 | 0.327 |
| Urban | 2706(88.5) | 2096(77.5) | 610(22.5) |  |  |
| Rural | 351(11.5) | 280(79.8) | 71(20.2) |  |  |
| Stage |  |  |  | 137.307 | <0.001 |
| Localized | 1152(37.7) | 796(69.1) | 356(30.9) |  |  |
| Regional | 835(27.3) | 624(74.7) | 211(25.3) |  |  |
| Distant | 1070(35.0) | 956(89.3) | 114(10.7) |  |  |
| Grade |  |  |  | 82.866 | <0.001 |
| Grade I | 37(1.2) | 11(29.7) | 26(70.3) |  |  |
| Grade II | 63(2.1) | 39(61.9) | 24(38.1) |  |  |
| Grade III | 1693(55.4) | 1278(75.5) | 415(24.5) |  |  |
| Grade IV | 1264(41.3) | 1048(82.9) | 216(17.1) |  |  |
