# Supplementary material for: Development and validation of nomograms to predict survival of neuroendocrine carcinoma in genitourinary system: A population-based retrospective study
Source: PLoS One. 2024 Jun 5;19(6):e0303440. doi: 10.1371/journal.pone.0303440 (PMC11152281; doi:10.1371/journal.pone.0303440)
Supplement: S3 Table — (DOCX) [file pone.0303440.s003.docx]

# S3 Table. Comparison of baseline characteristics of disease-specific survival (DSS) for patients with NEC.

| Variables | Total cohort | Disease-specific survival | | F/χ^2^ | P |
| --- | --- | --- | --- | --- | --- |
|  | (n=3057) | Dead from NEC  (n=1939) | Others  (n=1118) |  |  |
| Age | 69.0[57.0,78.0] | 69.00[57.0,79.0] | 68.00[56.3,76.0] | 3.022 | 0.003 |
| Sex |  |  |  | 2.028 | 0.154 |
| Male | 1654(54.1) | 1068(64.6) | 586(35.4) |  |  |
| Female | 1403(45.9) | 871(62.1) | 532(37.9) |  |  |
| Race |  |  |  | 0.001 | 0.992 |
| White | 2529(82.7) | 1604(63.4) | 925(36.6) |  |  |
| Others | 528(17.3) | 335(63.4) | 193(36.6) |  |  |
| System/Organ |  |  |  | 49.764 | <0.001 |
| Urinary System | 1650(54.0) | 985(59.7) | 665(40.3) |  |  |
| Bladder | 1525(49.9) | 914(59.9) | 611(40.1) |  |  |
| Kidney | 85(2.8) | 49(57.6) | 36(42.4) |  |  |
| Ureter | 40(1.3) | 22(55.0) | 18(45.0) |  |  |
| Female Genital System | 1026(33.6) | 653(63.6) | 373(36.4) |  |  |
| Uterus | 738(24.1) | 468(63.4) | 270(36.6) |  |  |
| Ovary | 219(7.2) | 138(63.0) | 81(37.0) |  |  |
| Vagina | 58(1.9) | 38(65.5) | 20(34.5) |  |  |
| Vulva | 11(0.4) | 9(81.8) | 2(18.2) |  |  |
| Male Genital System | 381(12.5) | 301(79.0) | 80(21.0) |  |  |
| Prostate | 378(12.4) | 300(79.4) | 78(20.6) |  |  |
| Testis | 2(0.1) | 0(0.0) | 2(100.0) |  |  |
| Penis | 1(0.0) | 1(100.0) | 0(0.0) |  |  |
| Pathology |  |  |  | 4.506 | 0.105 |
| SCNEC | 1856(60.7) | 1155(62.2) | 701(37.8) |  |  |
| LCNEC | 180(5.9) | 110(61.1) | 70(38.9) |  |  |
| NOS | 1021(33.4) | 674(66.0) | 347(34.0) |  |  |
| Surgery |  |  |  | 73.477 | <0.001 |
| None | 756(24.7) | 578(76.5) | 178(23.5) |  |  |
| Yes | 2301(75.3) | 1361(59.1) | 940(40.9) |  |  |
| Lymph node dissection |  |  |  | 69.998 | <0.001 |
| None | 2236(73.1) | 1517(67.8) | 719(32.2) |  |  |
| Yes | 821(26.9) | 422(51.4) | 399(48.6) |  |  |
| Radiotherapy |  |  |  | 0.417 | 0.519 |
| None/Unknown | 2070(67.7) | 1321(63.8) | 749(36.2) |  |  |
| Yes | 987(32.3) | 618(62.6) | 369(37.4) |  |  |
| Chemotherapy |  |  |  | 0.276 | 0.600 |
| None/Unknown | 1049(34.3) | 672(64.1) | 377(35.9) |  |  |
| Yes | 2008(65.7) | 1267(63.1) | 741(36.9) |  |  |
| Marital status |  |  |  | 1.680 | 0.195 |
| Married | 1780(58.2) | 1112(62.5) | 668(37.5) |  |  |
| Single | 1277(41.8) | 827(64.8) | 450(35.2) |  |  |
| Income |  |  |  | 0.480 | 0.488 |
| High | 1714(56.1) | 1078(62.9) | 636(37.1) |  |  |
| Low | 1343(43.9) | 861(64.1) | 482(35.9) |  |  |
| Residence |  |  |  | 0.562 | 0.453 |
| Urban | 2706(88.5) | 1710(63.2) | 996(36.8) |  |  |
| Rural | 351(11.5) | 229(65.2) | 122(34.8) |  |  |
| Stage |  |  |  | 217.683 | <0.001 |
| Localized | 1152(37.7) | 568(49.3) | 584(50.7) |  |  |
| Regional | 835(27.3) | 521(62.4) | 314(37.6) |  |  |
| Distant | 1070(35.0) | 850(79.4) | 220(20.6) |  |  |
| Grade |  |  |  | 43.591 | <0.001 |
| Grade I | 37(1.2) | 8(21.6) | 29(78.4) |  |  |
| Grade II | 63(2.1) | 29(46.0) | 34(54.0) |  |  |
| Grade III | 1693(55.4) | 1056(62.4) | 637(37.6) |  |  |
| Grade IV | 1264(41.3) | 846(66.9) | 418(33.1) |  |  |
