# Supplementary material for: Development and validation of nomograms to predict survival of neuroendocrine carcinoma in genitourinary system: A population-based retrospective study
Source: PLoS One. 2024 Jun 5;19(6):e0303440. doi: 10.1371/journal.pone.0303440 (PMC11152281; doi:10.1371/journal.pone.0303440)
Supplement: S4 Table — (DOCX) [file pone.0303440.s004.docx]

# S4 Table. Variable assignments.

| Variable | Risk Factors | Assignments |
| --- | --- | --- |
| X_1_ | Age | Continuous variable |
| X_2_ | Sex | Female=0, Male=1 |
| X_3_ | Pathology | No=0, Yes=1 |
| X_4_ | Surgery | No=0, Yes=1 |
| X_5_ | LND | No=0, Yes=1 |
| X_6_ | Radiotherapy | No=0, Yes=1 |
| X_7_ | Chemotherapy | No=0, Yes=1 |
| X_8_ | Marriage | No=0, Yes=1 |
| X_9_ | System=Urinary System | No=0, Yes=1 |
| X_10_ | System=Female Genital System | No=0, Yes=1 |
| X_11_ | System=Male Genital System | No=0, Yes=1 |
| X_12_ | Stage=Localized | No=0, Yes=1 |
| X_13_ | Stage=Regional | No=0, Yes=1 |
| X_14_ | Stage=Distant | No=0, Yes=1 |
| X_15_ | Grade=Grade I | No=0, Yes=1 |
| X_16_ | Grade=Grade II | No=0, Yes=1 |
| X_17_ | Grade=Grade III | No=0, Yes=1 |
| X_18_ | Grade=Grade IV | No=0, Yes=1 |
