# Supplementary material for: Development and validation of nomograms to predict survival of neuroendocrine carcinoma in genitourinary system: A population-based retrospective study
Source: PLoS One. 2024 Jun 5;19(6):e0303440. doi: 10.1371/journal.pone.0303440 (PMC11152281; doi:10.1371/journal.pone.0303440)
Supplement: S5 Table — (DOCX) [file pone.0303440.s005.docx]

# S5 Table. Risk factors selected by LASSO.

| Variable | Risk Factors | Coefficient | Odds ratio |
| --- | --- | --- | --- |
| **Overall survival** | | | |
| X_1_ | Age | 0.012 | 1.012 |
| X_4_ | Surgery | -0.016 | 0.984 |
| X_5_ | LND | -0.349 | 0.705 |
| X_7_ | Chemotherapy | -0.231 | 0.794 |
| X_12_ | Stage=Localized | -0.183 | 0.833 |
| X_14_ | Stage=Distant | 0.513 | 1.670 |
| X_15_ | Grade=Grade I | -0.181 | 0.835 |
| X_16_ | Grade=Grade II | -0.256 | 0.774 |
| **Disease-specific survival** | | | |
| X_1_ | Age | 0.007 | 1.007 |
| X_4_ | Surgery | -0.027 | 0.973 |
| X_5_ | LND | -0.299 | 0.742 |
| X_7_ | Chemotherapy | -0.136 | 0.873 |
| X_12_ | Stage=Localized | -0.263 | 0.769 |
| X_14_ | Stage=Distant | 0.528 | 1.696 |
| X_15_ | Grade=Grade I | -0.062 | 0.940 |
| X_16_ | Grade=Grade II | -0.164 | 0.849 |
