# Supplementary figures and images for: Development and validation of nomograms to predict survival of neuroendocrine carcinoma in genitourinary system: A population-based retrospective study
Source: PLoS One. 2024 Jun 5;19(6):e0303440. doi: 10.1371/journal.pone.0303440 (PMC11152281; doi:10.1371/journal.pone.0303440)

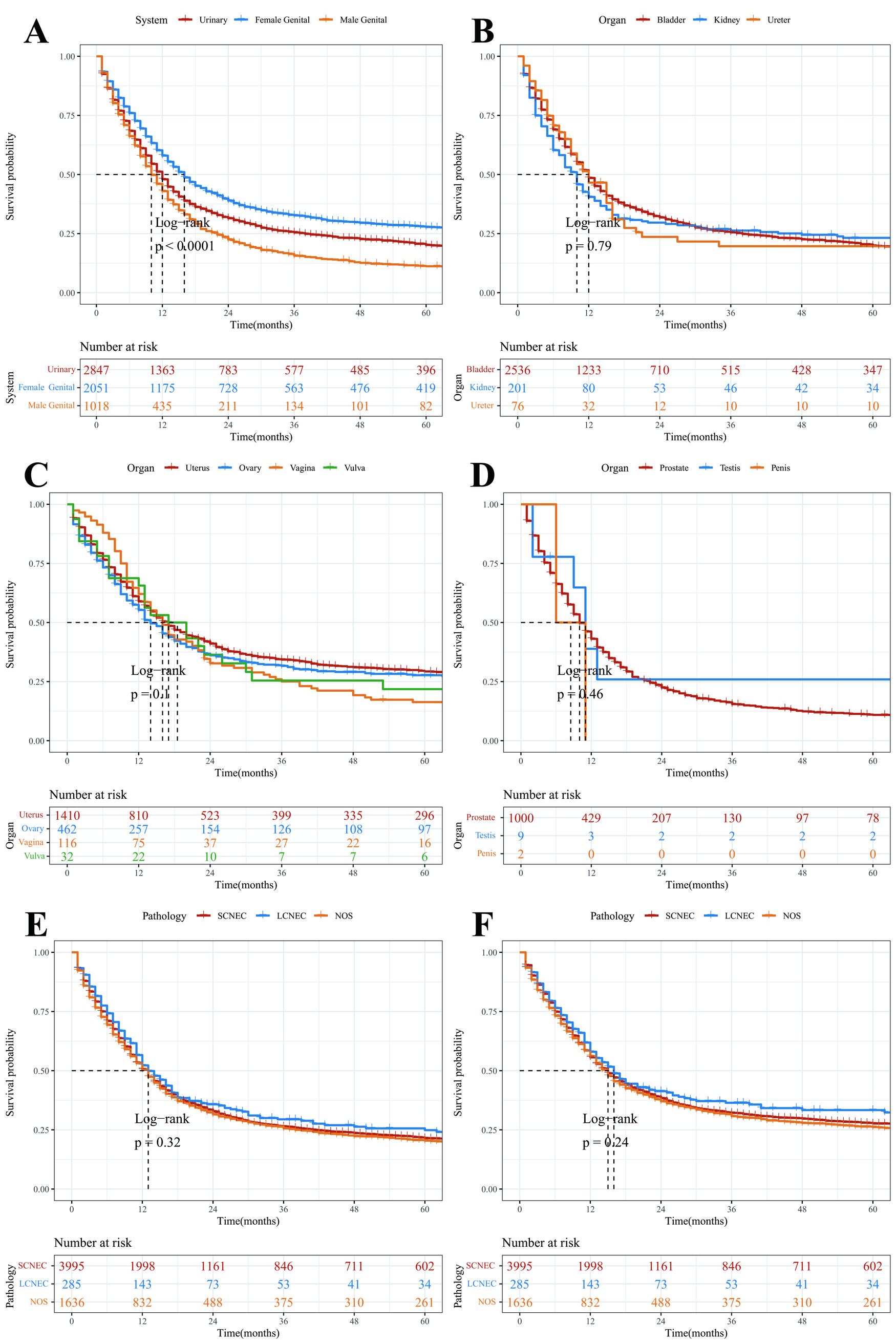

Supplement: S1 Fig — (TIF) [file pone.0303440.s006.tif]

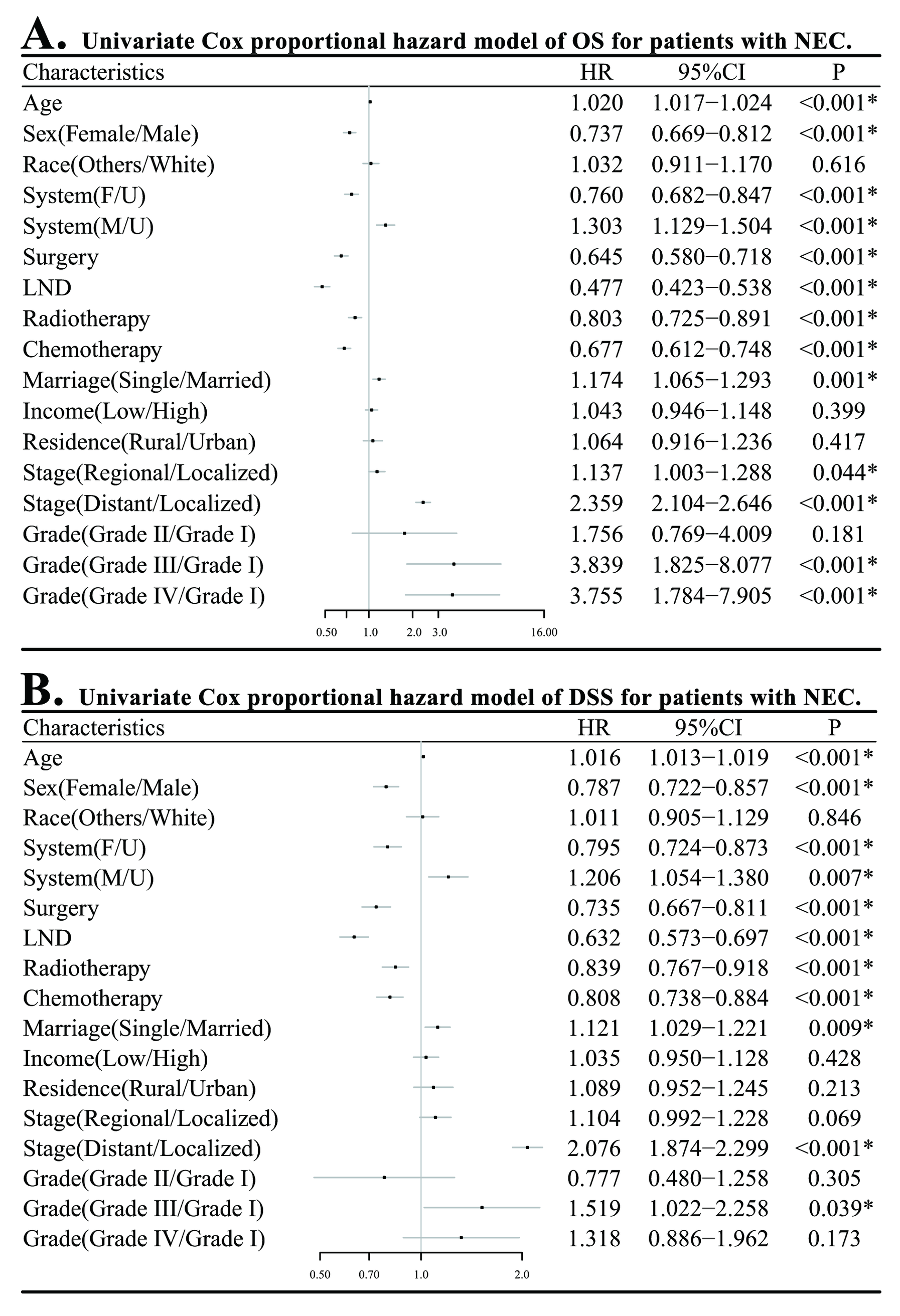

Supplement: S2 Fig — (TIF) [file pone.0303440.s007.tif]

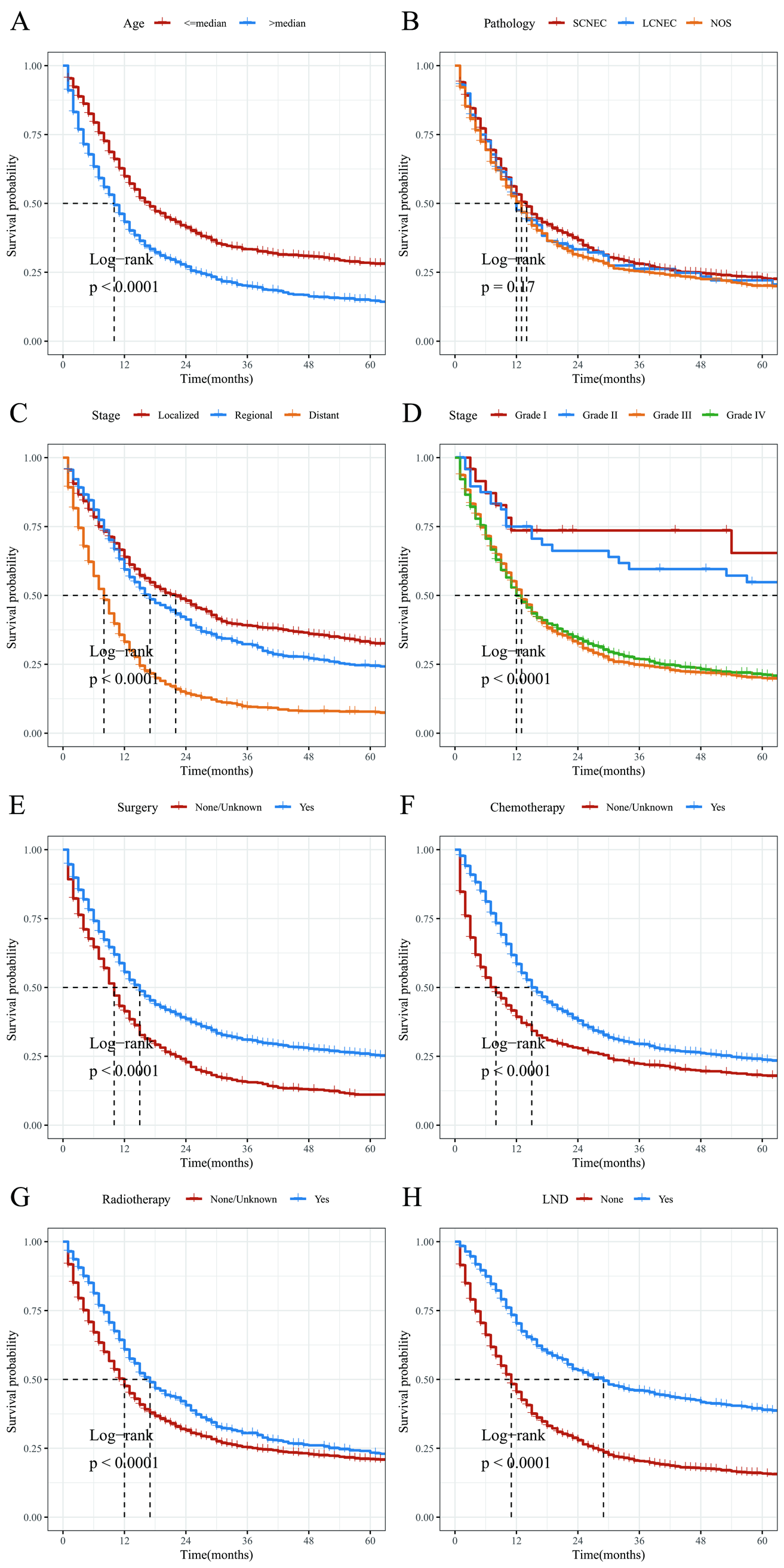

Supplement: S3 Fig — (TIF) [file pone.0303440.s008.tif]

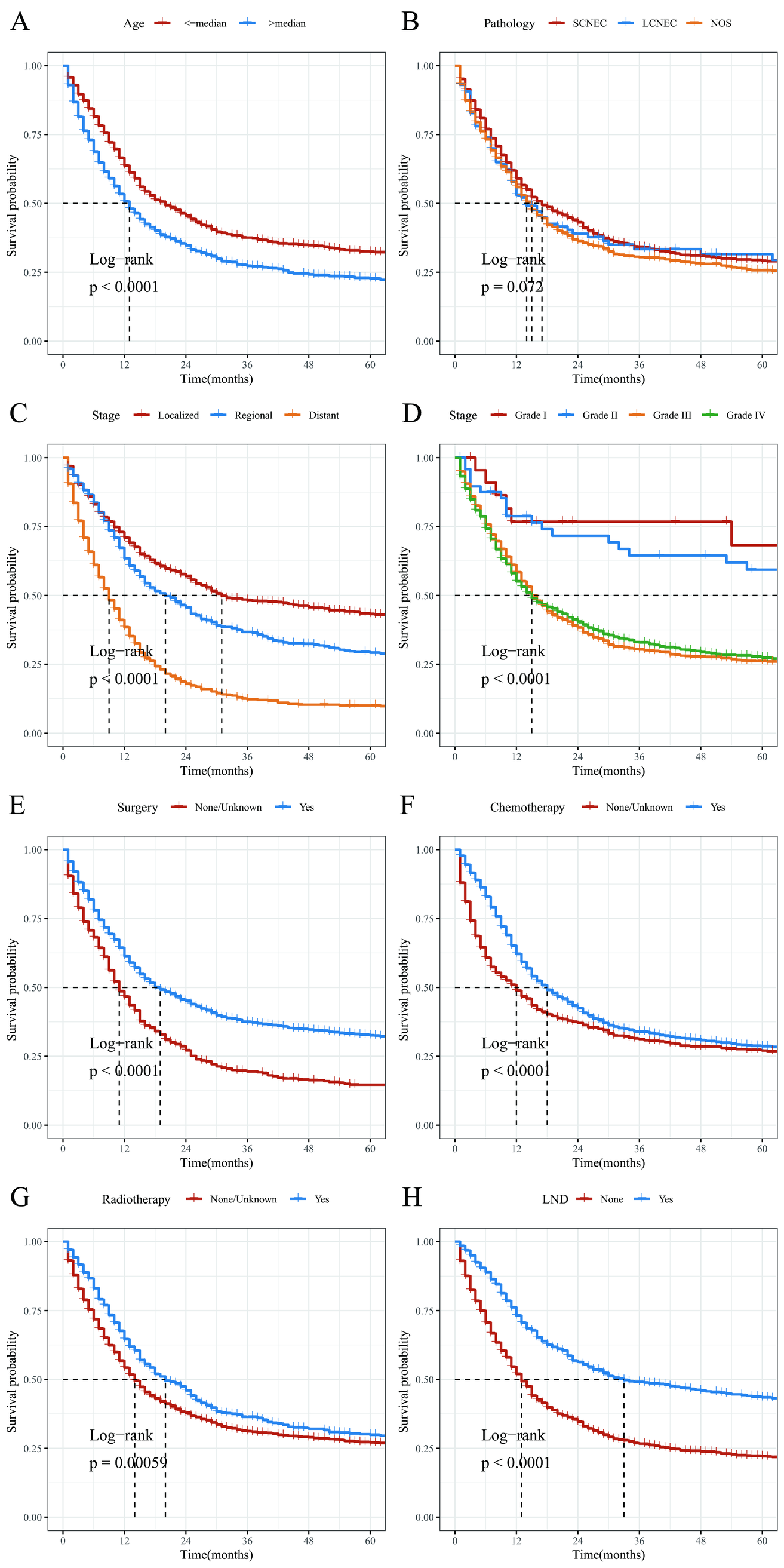

Supplement: S4 Fig — (TIF) [file pone.0303440.s009.tif]

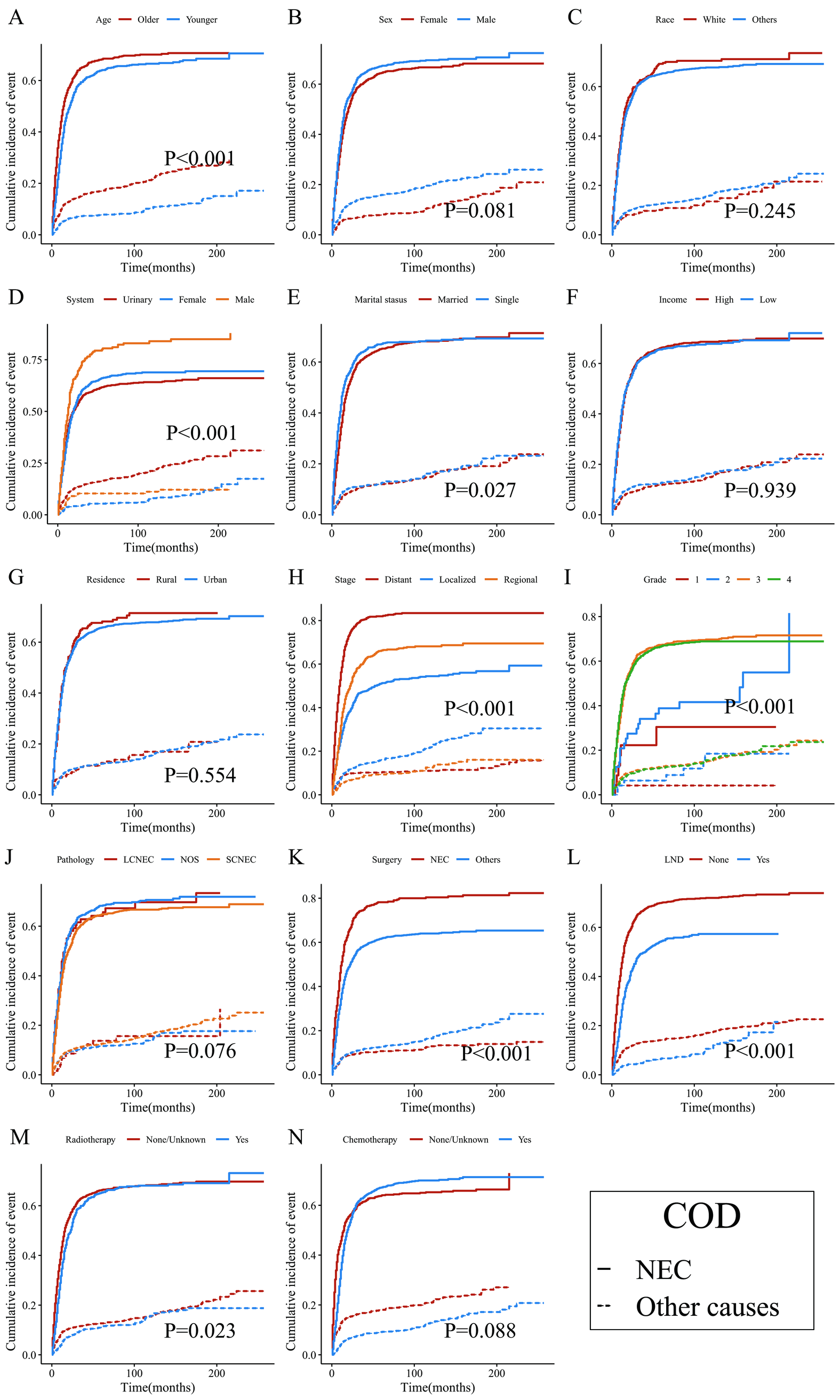

Supplement: S5 Fig — (TIF) [file pone.0303440.s010.tif]

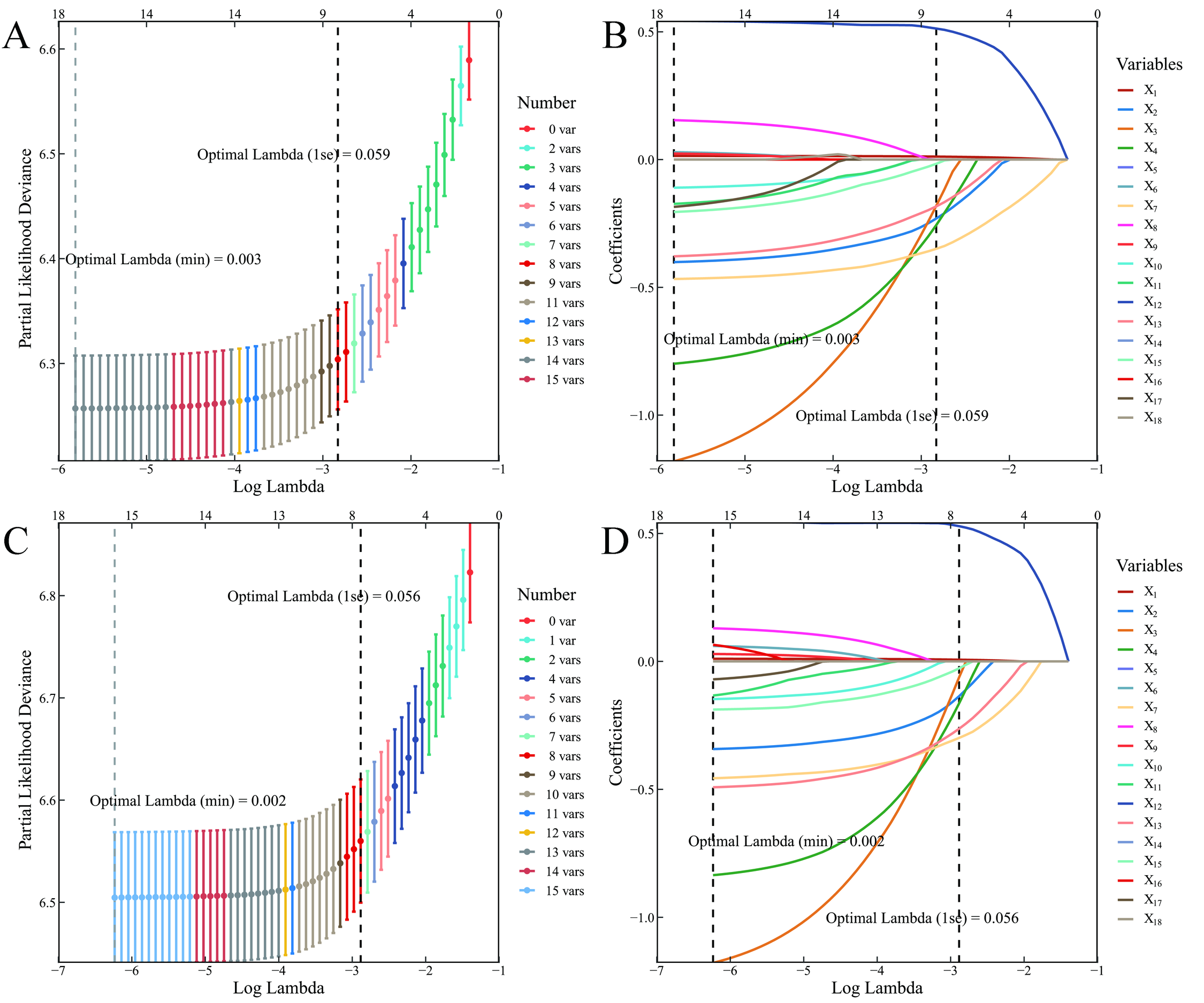

Supplement: S6 Fig — (TIF) [file pone.0303440.s011.tif]
